# Short-term nutritional counseling reduces body mass index, waist circumference, triceps skinfold and triglycerides in women with metabolic syndrome

**DOI:** 10.1186/1758-5996-2-13

**Published:** 2010-02-10

**Authors:** Gustavo D Pimentel, Silvia T Arimura, Bruno M de Moura, Maria ER Silva, Maysa V de Sousa

**Affiliations:** 1 Department of Physiology, Federal University of São Paulo (UNIFESP), São Paulo/SP, Brazil; 2 Nutrition Course, Mogi das Cruzes University (UMC), Mogi das Cruzes/SP, Brazil; 3 Laboratory of Medical Investigation (LIM 18), Endocrinology, School of Medicine (USP), São Paulo/SP, Brazil

## Abstract

**Background:**

It is recognized that the growing epidemic of metabolic syndrome is related to dietary and lifestyle changes.

**Objective:**

The purpose of this study was to evaluate short-term application of nutritional counseling in women with metabolic syndrome.

**Methods:**

This follow-up study was conducted from September to November 2008 with thirty three women ≥35 years old screened clinically for nutritional counseling. Dietary intake was reported, and biochemical and body composition measures were taken at baseline and after three months of follow-up.

**Results:**

Of the 33 women evaluated, 29 patients completed the study. The prevalence of type 2 diabetes mellitus, hypertension, dyslipidemia, and obesity was high at 38%, 72.4%, 55.2%, and 75.8%, respectively. At the end of three-months of follow-up, a significant decline in body mass index, waist circumference, triceps skinfold, and triglycerides was observed, as was an increase in calcium and vitamin D intake. The multiple regression analysis showed that changes in body mass index, triceps skinfold, waist circumference and triglyceride levels after nutritional intervention were positively associated with changes in anthropometric (loss of body weight) and biochemical (decrease of TG/HDL-c ratio) parameters. Moreover, waist circumference changes were negatively associated with changes in calcium and vitamin D intake.

**Conclusion:**

Short-term nutritional counseling improved some factors of metabolic syndrome. Moreover, the increases in calcium and vitamin D consumption can be associated with the improvement in markers of metabolic syndrome.

## Introduction

Metabolic syndrome was defined in 2001 by the National Cholesterol Education Program Adult Treatment Panel III (ATP III) as the presence of ≥ 3 of the following risk factors: abdominal obesity (high waist circumference), hyperglycemia, hypertriacylglycerolemia, low HDL-c, and hypertension [1].

The high prevalence of metabolic syndrome in developing countries have been demonstrated in countries such as: Morocco, Oman, Turkey, Iran with 33.5%, 16.3%, 33.4%, and 33.7%, respectively. In South America the prevalence is 31.2% in Venezuela and 25.4% in Brazil [2].

It is recognized that the growing epidemic of chronic diseases is related to dietary and lifestyle changes in the post-industrial era. Several studies have demonstrated the association between nutrition and obesity, coronary heart disease, diabetes, osteoporosis, and certain cancers (for review see WHO [3]). In this case, the prevention of metabolic syndrome represents an important opportunity to ameliorate the health of these patients. Thus the Dietary Guidelines for Americans [4], the Brazilian Guidelines on Diagnosis and Treatment of Metabolic Syndrome [5], and long-term lifestyle intervention [6-8] and short-term [9] emphasizes low consumption of hypercaloric food, recommends increased amounts of whole grain, fruit and vegetables, and a limited intake of *trans *and saturated fatty acids.

The purpose of this study was to evaluate the effects of short-term application of nutritional counseling in Brazilian women with metabolic syndrome.

## Methods

### Subjects and Methods

**Figure 1:**
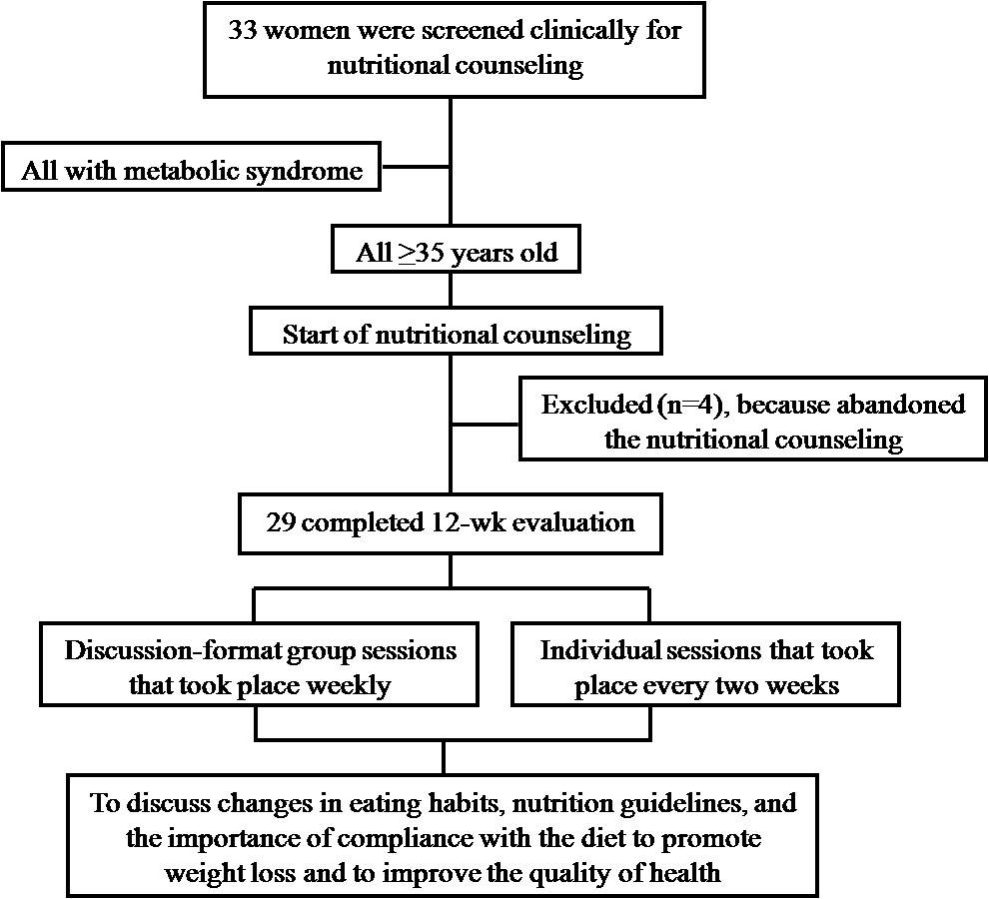
**Flowchart of participation in the study**.

This follow-up study was conducted from September to November 2008 in patients screened clinically for nutritional counseling. The sample consisted of women attended by the Family Health Program of Mogi das Cruzes, located in the Metropolitan Area of São Paulo, Brazil. The only criterion for inclusion was women with metabolic syndrome (defined by the ATP III), but women with liver, kidney or heart diseases were excluded, as were women with chronic consumption of alcohol. As a whole, 33 women from 35 to 77 years old were evaluated (Figure 1). All the participants signed the prior informed consent designed according to the n° 196/96 on "Research involving human beings, from the Health Board of the Ministry of Health" approved by the Ethics Committee of Mogi das Cruzes University, under number 131/2008.

### Anthropometry and body composition assessments

Body weight was obtained to the nearest 0.01 kg with an electronic scale (Filizola^®^, São Paulo, SP, Brazil) and height was measured to the nearest 0.5 cm without shoes while standing on a level, hard surface using a calibrated stadiometer (Sanny^®^, Brazil) followed by body mass index (BMI) calculation [10] and according to the norms standard by Heyward & Stolarczyk [11].

The waist circumference was measured 2 cm over the umbilical scar [12] and arm circumference was measured to evaluate the reduction of the muscle or fat mass from reduction of body weight. This was followed by evaluations of triceps and biceps skinfold thickness, measured at the midpoint of the upper arm and was expressed in mm by means of a Lange skinfold caliper in triplicate [13].

### Nutritional counseling

Participants received individual and group counseling with a team of nutritionists. The dietary intervention consisted of discussion-format group sessions that took place weekly and individual sessions that took place every two weeks to discuss changes in eating habits, nutrition guidelines, and the importance of compliance with the diet to promote weight loss and to improve the quality of health (Figure 1). In other words, the topics discussed were about the importance of healthy eating (increasing intake of fruits, vegetables, fish and water and reducing consumption of sugar, fat, sodium, and fried foods) to diminish the components of metabolic syndrome.

The intervention included written and oral instructions in the form of lecture, with the aid of a sequence of posters. Patients were also encouraged to increase physical activity, mainly by walking, swimming or aerobic ball games.

### Dietary measurements

Initial and follow-up visits included anthropometrical data, biochemical parameters, as well as assessment of current diet.

The 24 h recall questionnaire was performed before and after nutritional counseling and was evaluated for the presence or absence of changes in eating habits. The food frequency questionnaire (FFQ) only was available at the baseline point. Nutritional analysis was carried out with the software NutWin^® ^(Federal University of São Paulo, Brazil). Table of Food Composition-Brazil [14] and TACO version 2-Brazil [15] tables were used to determine the macro and micronutrients content of foods.

### Sociodemographic characteristics

The women answered questionnaires (baseline moment) with regard to sociodemographic factors. Subjects also were asked about home conditions, basic sanitation, health-related habits (smoking status, alcohol intake, dieting in the past, and whether they are physically active or not). Type 2 diabetes mellitus, hypertension, dyslipidemia and obesity were defined by Brazilian Guidelines on Diagnosis and Treatment of Metabolic Syndrome [5].

### Biochemical measurements

Blood samples were obtained after a 12-hour fast and analyzed in the Hospital Laboratory of the city of Mogi das Cruzes, São Paulo, Brazil. Labtest^® ^kits were used to assess fasting blood glucose, total cholesterol, HDL-c and triglycerides. The samples were analyzed using an enzymatic method. LDL-c was calculated according to the Friedwald equation (LDL-c = total cholesterol-(HDL-c)-(TG/5) [16] and LDL-c subclass by the equation (TG/HDL-c ratio) [17] which is a good predictive factor for oxidized-LDL-c.

### Statistical analysis

Implementation of the Kolmogorov-Smirnov test revealed that the results of experiments were distributed normally. Thus, the variables were expressed by descriptive analysis (mean and standard deviation) and a t-test was used to check for any possible differences between findings at the baseline and after nutritional counseling.

Linear relationships were estimated using Pearson correlation (crude model) to examine the associations between changes in variables, expressed as percentage of change ((endpoint - baseline value)/baseline value × 100). Moreover, a multiple regression analysis was performed to identify the contribution of changes in body composition, food intake and biochemical measurements to changes in metabolic parameters independent of other factors. Thus, three models of adjustment were performed, first adjusted by age (model 1), adjusted by BMI (model 2) and adjusted by total calories intake (model 3).

The level of statistical significance used was 5% in all tests. For all analyses we used software STATISTICA version 6.0.

## Results

Of the 33 women evaluated, 29 patients completed the study. Four participants abandoned the nutritional counseling (Figure 1).

The dietetic, anthropometric, and biochemical characteristics at baseline and after three months of nutritional counseling are shown in Table 1. At the end of three months of follow-up, a significant decline in BMI, waist circumference, triceps skinfold, and triglycerides was observed, as well as an increase in calcium and vitamin D intake. The total calorie and macronutrient intake didn't alter after nutricional counseling (Table 1). Sociodemographic characteristics of the participants (Table 2) demonstrated that most were living in owned houses (96.5%), had basic sanitation (96.5%), didn't smoke or drink alcohol (82.8% and 90.5%), didn't try to lose weight by dieting in the past (65.5%), and were sedentary (79.3%). The prevalence of type 2 diabetes mellitus, hypertension, dyslipidemia, and obesity was high, at 38%, 72.4%, 55.2%, and 75.8%, respectively.

**Table 1:** Anthropometric, dietetic, and biochemical variables at baseline and after nutritional counseling.

Variables	Baseline	After nutritional counseling	p value
Age (y)	56.7 ± 9.0	-	-
Body mass index (kg/m^2^)	33.6 ± 6.0	33.1 ± 5.7	0.009*
Waist circumference (cm)	104.8 ± 11.6	101.9 ± 11.0	0.0008*
Biceps skinfold (mm)	35.5 ± 3.8	35.3 ± 4.1	0.41
Triceps skinfold (mm)	36.0 ± 8.5	34.2 ± 8.2	0.031*
Calories (kcal)	1140.0 ± 287.3	1210.7 ± 353.6	0.288
Carbohydrate (%)	55.1 ± 12.2	59.5 ± 12.0	0.178
Protein (%)	20.9 ± 6.0	18.4 ± 4.9	0.084
Lipids (%)	23.9 ± 10.7	22.0 ± 9.8	0.518
Dietary fiber (g)	9.4 ± 4.9	9.5 ± 7.0	0.982
Calcium (mg)	423.3 ± 252.8	587.3 ± 251,3	0.023*
Phosphor (mg)	694.0 ± 271.0	762.6 ± 284.0	0.240
Sodium (mg)	1965.0 ± 892.3	1844.4 ± 639.4	0.541
Magnesium (mg)	193.2 ± 97.7	191.5 ± 66.2	0.940
Potassium (mg)	2241.6 ± 1026.2	2332.8 ± 867.7	0.717
Vitamin D (mg)	1.8 ± 1.0	2.8 ± 1.7	0.013*
Fasting glucose (mg/dL)	120.8 ± 37.0	107.5 ± 34.8	0.133
Total cholesterol (mg/dL)	217.3 ± 54.9	196.8 ± 41.2	0.112
HDL-c (mg/dL)	47.0 ± 9.1	49.9 ± 10.7	0.194
LDL-c (mg/dL)	137.0 ± 52.1	118.8 ± 42.0	0.125
Triglycerides (mg/dL)	168.0 ± 56.2	140.6 ± 65.0	0.045*
TG/HDL-c ratio	3.7 ± 1.5	3.1 ± 2.0	0.155

BMI: body mass index; HDL-c: high density lipoprotein; LDL-c: low density lipoprotein.

*p < 0.05 significative difference versus basal.

**Table 2:** Sociodemographic characteristics of the participants.

Variables	n (%)
**Home**	
Rented	1 (3.5)
Own house	28 (96.5)
**Basic sanitation**	
Yes	28 (96.5)
No	1 (3.5)
**Smoke**	
Yes	5 (17.2)
No	24 (82.8)
**Alcoholic drink**	
Yes	3 (9.5)
No	26 (90.5)
**Dieting in the past**	
Yes	10 (34.5)
No	19 (65.5)
**Physical activity**	
Yes	6 (20.7)
No	23 (79.3)
**Diabetes**	
Yes	11 (38)
No	18 (62.0)
**Hypertension**	
Yes	21 (72.4)
No	8 (27.6)
**Dyslipidemia**	
Yes	16 (55.2)
No	13 (44.8)
**Obesity**	
Yes	22 (75.8)
No	7 (24.2)

At the baseline, FFQ showed that all (100%) subjects consumed rice, breads or roots daily, fruits (55% daily, 41.5% weekly, and 3.5% monthly) and vegetables (72.5% daily, 13.8% weekly, 3.5% monthly, and 10.2% never), meat and eggs (79.3% daily and 20.7% weekly), beans (93.0% daily and 7.0% weekly), oil and fats (62.0% daily, 24.0% weekly, 3.5% monthly, and 10.5% never) and sugar and sweets (48.2% daily, 24.0% weekly, 10.3% monthly, and 17.5% never). Furthermore, Pearson's correlation demonstrated that biceps skinfold was associated positively with meat serving (r = 0.56), triceps skinfold with meat serving (r = 0.59) and vegetable oil serving (r = 0.53).

In the studied individuals, BMI, triceps skinfold, waist circumference and triglycerides changes were positively associated with body weight, BMI, waist circumference, and TG/HDL-c ratio (all Pearson correlation coefficients >0.42; all p values ≤ 0.05 or ≤ 0.0001). Besides, waist circumference were negatively associated with calcium (r = -0.37, p ≤ 0.05) and vitamin D (r = -0.39, p ≤ 0.05) intake, suggesting that both increases were linked to the reduction of abdominal fat. The triglycerides changes was also inversely (r = -0.86, p ≤ 0.0001) associated with changes in HDL-c concentrations (Table 3).

**Table 3:** Partial correlation of changes (Δ percentage) in body mass index, triceps skinfold, waist circumference measurements and triglycerides concentrations after three months related to changes in metabolic parameters adjusted for age, body composition and calories intake.

Variables	Crude				Model 1				Model 2			Model 3			
	
	BMI	TSF	WC	TG	BMI	TSF	WC	TG	TSF	WC	TG	BMI	TSF	WC	TG
Body weight	**0.96‡**	0.16	0.38	-0.45	**0.96‡**	-0.13	-0.16	-0.03	**0.97‡**	**0.96‡**	**0.97‡**	-0.12	-0.18	-0.12	-0.03

Body mass index	-	0.19	**0.42†**	-0.46	-	-0.09	-0.09	0.11	-	-	-	-	-0.18	-0.10	0.11

Waist circumference	**0.42†**	0.00	-	-0.27	-0.09	-0.20	-0.16	-0.16	0.42	-	0.41	-0.10	-0.03	-	-0.16

Biceps skinfold	0.26	0.11	0.35	-0.27	0.11	-0.11	-0.06	-0.25	0.08	0.34	0.62	-0.20	0.14	0.11	-0.25

Triceps skinfold	0.19	-	0.00	-0.07	0.18	-	-0.20	0.21	-	0.42	-0.03	-0.18	-	-0.03	0.21

Glucose	0.20	0.52	-0.40	-0.46	0.06	0.60	-0.12	-0.27	-0.02	0.45	0.20	0.03	0.32	0.34	-0.27

Cholesterol	0.23	-0.29	0.17	-0.10	0.11	0.21	-0.16	-0.21	-0.05	0.08	0.01	0.03	0.35	0.35	-0.08

HDL-c	0.37	0.13	0.36	**-0.86‡**	0.43	0.21	-0.16	-0.31	-0.05	0.41	0.38	0.03	0.35	0.35	-0.31

LDL-c	0.25	-0.44	0.18	-0.19	0.11	-0.42	-0.16	-0.17	-0.05	0.41	0.25	-0.01	0.35	0.35	-0.16

Triglycerides	-0.46	-0.07	-0.27	-	0.11	0.21	-0.16	-0.17	-0.03	-0.09	-	-0.01	0.04	0.40	-

TG/HDL-c ratio	-0.46	0.02	-0.24	**0.98‡**	-0.49	0.21	-0.16	0.13	-0.00	0.41	**0.97‡**	-0.01	0.35	0.35	0.13

Calories	-0.10	0.32	0.15	0.22	-0.09	0.32	0.15	0.13	0.19	**0.42†**	-0.46	-	-	-	-

Carbohydrate	0.01	-0.25	-0.20	-0.34	-0.09	-0.05	-0.11	0.13	0.19	-0.23	-0.46	-0.10	0.32	0.15	0.22

Protein	0.06	-0.12	0.17	-0.15	0.10	-0.05	-0.11	0.13	-0.07	**0.42†**	0.06	-0.10	0.32	0.15	0.22

Lipids	-0.07	0.23	0.11	0.31	-0.09	0.22	-0.11	0.13	0.19	**0.42†**	-0.46	-0.10	0.32	0.15	0.22

Dietary fiber	-0.13	-0.01	0.12	-0.17	-0.09	-0.05	-0.11	0.13	0.19	0.18	-0.46	-0.10	0.32	0.15	0.22

Calcium	-0.18	-0.22	**-0.39†**	0.12	-0.17	-0.05	-0.11	0.13	0.19	**0.42†**	-0.07	-0.10	0.32	0.15	0.22

Phosphoro	-0.08	-0.13	0.01	-0.19	-0.09	-0.12	0.03	0.13	0.19	**0.42†**	-0.07	-0.10	0.32	0.15	0.22

Sodium	-0.19	0.34	-0.26	0.06	-0.09	-0.05	-0.11	0.13	0.19	**0.42†**	-0.07	-0.10	0.32	0.15	0.22

Magnesium	-0.22	-0.22	-0.11	0.33	-0,21	-0.05	-0.11	0.13	0.19	**0.42†**	0.27	-0.10	0.32	0.15	0.22

Potassium	-0.12	-0.30	-0.11	0.24	-0.09	-0.05	-0.11	0.13	0.19	**0.42†**	-0.46	-0.10	0.32	0.15	0.22

Vitamin D	-0.03	-0.16	**-0.37†**	0.19	-0.09	-0.05	-0.11	0.13	0.19	**0.42†**	-0.46	-0.10	0.32	0.15	0.22

Multiple regression analysis was performed to identify the contribution of changes in body composition, food intake and biochemical measurements to changes in metabolic parameters independent of other factors. The value were expressed as percentage of change ((endpoint - baseline value)/baseline value × 100). Crude model: withouth adjustment, Model 1: adjusted by age, Model 2: adjusted by BMI and Model 3: adjusted by calories intake. BMI: body mass index, TSF: triceps skinfold, WC: waist circumference, TG: triglycerides.

†p ≤ 0.05, ‡p ≤ 0.0001

The multiple regression analysis adjusted by age (model 1) showed a positive correlation between BMI and body weight (r = 0.96, p ≤ 0.0001). When adjusted by BMI (model 2), we observed a positive correlation between triceps skinfold, waist circumference and triglycerides with body weight, TG/HDL-c ratio, total calories, proteins, lipids, calcium, phosphorus, magnesium, potassium and vitamin D intake (all correlations r > 0.42, for all values of p ≤ 0.05 or ≤ 0.0001).

In order to verify whether the caloric intake may interfere with the changes to the anthropometric and triglycerides levels, a multiple regression analysis was performed adjusted by energy intake (model 3). It could be observed that the decrease in triglycerides and anthropometric indicators were influenced by the total caloric intake, because after adjustment by calorie intake (model 3) significant losses were observed in all correlation performed (Table 3).

## Discussion

In this study, we tested the hypothesis that a nutritional counseling approach aimed at reducing BMI, waist circumference, triceps skinfold and triglycerides and increasing calcium and vitamin D intake is effective over a short-term (three months) at reducing markers of metabolic syndrome. Regarding the Pearson correlation and multiple regression, we showed that the reduction in BMI, waist circumference, triceps skinfold, and triglycerides can be associated with the decrease in body weight and TG/HDL-c ratio, as well as with the increases in calcium and vitamin D intake.

Several studies have revealed the importance of nutritional education aiming at preventing metabolic syndrome [6,7,9]. The present study confirmed this by observing a significant decline in BMI, triceps skinfold and triglycerides and increases in calcium and vitamin D intake. This increase in calcium and vitamin D may have occurred by substitution of milk, cheese and butter for low fat dairy products observed during nutritional counseling (data not published) which is rich in calcium and vitamin D.

Calcium is one major nutritional component in dairy products. Dietary calcium may lower the activity of renin-angiotensin system, improve sodium-potassium balance, and inhibit vascular smooth muscle cell constriction [18]. Moreover, high calcium intake facilitates weight loss and enhances insulin sensitivity, which also contribute to blood pressure reduction [19].

Recently, numerous interventional and epidemiological [20-25] studies have shown that the calcium and vitamin D consumption are associated with and effectively reduce abdominal fat and metabolic syndrome risk.

Similar to previous studies [26-28] we found that abdominal fat is considered one of the strongest indicators of metabolic syndrome and is associated with TG/HDL-c ratio.

In the present study, we evaluated the predictive factor for oxidized-LDL-c by using the equation (TG/HDL-c ratio) [17], but didn't observe a reduction after nutritional counseling. Obviously, the TG/HDL-c ratio was associated with triglycerides concentrations, suggesting that reductions in triglycerides also diminished TG/HDL-c ratio. In this context, several studies have demonstrated that oxidized-LDL-c activates circulating monocytes, increasing their ability to infiltrate the vascular wall and this increased infiltration is a primary stage for atherogenesis [29,30] and worsening of metabolic syndrome. The oxidized-LDL-c increases triglycerides production by inducing the accumulation of fatty acids in adipocytes [31]. Moreover, hypertriglyceridemia is associated with higher levels of small dense LDL-c, which is particularly prone to oxidation and has been proven to be more atherogenic than larger LDL-c particles [32,33]. Fortunately, in the present study we managed to lower the triglyceride levels at the end of study.

As mentioned previously, the women in this study had little change in their BMI but showed a decrease in waist circumference. In fact, the nutritional counseling may have modest effects on decreasing BMI and more effect on abdominal fat loss. It has already been established that the waist circumference is positively associated with all the obesity-related abnormalities, such as hypertension, dyslipidemia, diabetes, metabolic syndrome, cardiovascular diseases, stroke and myocardial infaction [34,35].

Waist circumference and skinfold measurements can be used to assess body composition and are cheaper than imaging technique such as dual-energy X-ray absorptiometry, air-displacement plethysmography or hydrostatic weighing that may provide a more accurate method for assessing body composition [36,37] and the metabolic syndrome risk.

Our study has some limitations. First of all, the Brazilian population has a high degree of miscegenation that includes a mix of indigenous people, Afro- and Euro-Brazilians, and a widespread Latin ancestry. Thus, we do not know if this genetic and environmental diversity can modify the relationship between nutritional counseling and diminishing metabolic syndrome factors. Second, the low number of participants included in study. Third, the short-term of the nutritional counseling, lasting only three months. Fourth, the lack of a control group.

The lifestyle modifications that combine energy restriction and healthy eating (increased intake of fruits, vegetables, fish and water and reduced consumption of sugar, fat, sodium, and fried foods) would appear to be a preferred treatment strategy for metabolic syndrome. However, these results are limited to overweight and obese women with metabolic syndrome attended by the Family Health Program in a little city of the State of São Paulo, Brazil. Moreover, the subjects were verbally informed to do physical activity. However, it is not known if everyone started physical activity and whether this could be influencing the results.

Therefore, further research is required to assess the benefits of nutritional counseling in both women and men with metabolic syndrome. Additional studies are also required to evaluate the individual effects of different nutrients and their long-term effects.

In summary, this study shows that the reduction of metabolic syndrome factors can be effectively achieved by short-term nutritional counseling. The lifestyle interventions proposed reduces BMI, waist circumference, triceps skinfold, and triglycerides concentrations. Moreover the reduction in BMI, waist circumference, triceps skinfold and triglycerides was partially associated with the decrease in body weight and TG/HDL-c ratio, as well as with the increases in calcium and vitamin D intake, independent of age and body composition. However, further studies are warranted to confirm the potential benefit of nutritional counseling in mitigating metabolic syndrome.

## Competing interests

The authors declare that they have no competing interests.

## Authors' contributions

GDP performed the statistical analysis and written of article. STA and BMM has collected the data and carried out the nutritional counseling. MERS and MVS conceived of the study, participated in its design and coordination. All authors read and approved the final manuscript.
